# A Cross-sectional Analysis of 556 Eyes Entering the Homburg Aniridia Centre

**DOI:** 10.1055/a-2065-8405

**Published:** 2023-08-30

**Authors:** Fabian Norbert Fries, Annamária Náray, Cristian Munteanu, Tanja Stachon, Neil Lagali, Berthold Seitz, Nóra Szentmáry, Barbara Käsmann-Kellner

**Affiliations:** 1 Dr. Rolf M. Schwiete Center for Limbal Stem Cell and Aniridia Research, Saarland University, Homburg/Saar, Germany; 2 Department of Ophthalmology, Saarland University Medical Center, Homburg/Saar, Germany; 3 Department of Ophthalmology, Semmelweis University, Budapest, Hungary; 4 Department of Biomedical and Clinical Sciences, Faculty of Medicine, Linköping University, Linköping, Sweden

**Keywords:** congenital aniridia, Homburg Aniridia Center, glaucoma, macular hypoplasia, aniridia-associated keratopathy, Pax-6 gene, kongenitale Aniridie, Pax-6-Gen, Glaukom, Makulahypoplasie, Homburger Aniridiezentrum, Aniridie-assoziierte Keratopathie

## Abstract

**Purpose** Congenital aniridia is a severe malformation of almost all eye segments. In addition, endocrinological, metabolic, and central nervous systems diseases may be present. In order to develop better treatment options for this rare disease, an aniridia center must be established. The purpose of this work is to summarize ophthalmic findings of aniridia subjects examined at the Department of Ophthalmology, Saarland University Medical Center in Homburg.

**Methods** Our retrospective single-center study included patients who underwent a comprehensive ophthalmic examination through the head of the KiOLoN (“Kinderophthalmologie”, Orthoptics, Low Vision and Neuroophthalmology) Unit of the department between June 2003 and January 2022. Data at the first examination time point have been included.

**Results** Of 286 subjects, 556 eyes of (20.1 ± 20.1 years; 45.5% males) were included. There was nystagmus in 518 (93.7%) eyes, and strabismus in 327 (58.8%) eyes. There were 436 (78.4%) eyes with age-appropriate axial length, 104 (18.7%) eyes with microphthalmos, and 13 (2.3%) eyes with buphthalmos. There was iris malformation with atypical coloboma in 34 eyes (6.1%), more than 6 clock hours of iris remnants in 61 eyes (10.9%), less than 6 clock hours of iris remnants in 96 eyes (17.2%), and complete aniridia in 320 (57.5%) eyes. The patients were graded according to the following aniridia-associated keratopathy (AAK) stages: Stage 0 (96 eyes [17.2%], no keratopathy), Stage 1 (178 eyes [32.0%]), Stage 2 (107 eyes [19.2%]), Stage 3 (67 eyes [12.0%]), Stage 4 (62 eyes [11.1%]), Stage 5 (45 eyes [8.0%]). There was secondary glaucoma in 307 (55.5%), macular hypoplasia in 395 (71.4%), and congenital optic nerve head pathology in 223 (40.3%) eyes. The iris malformation type was significantly positively correlated with AAK stage, lens properties, presence of glaucoma, congenital macular, and optic nerve head properties (p < 0.001 for all), while complete aniridia showed the most complications.

**Conclusions** At the Homburg Aniridia Center, the most common ophthalmic signs in congenital aniridia were AAK, iris malformation, cataract, and macular hypoplasia. The iris malformation type may indicate future expression of AAK, cataract, and glaucoma development and it is correlated with a congenital optic nerve head and macular pathology. Our registry will support further detailed longitudinal analysis of ophthalmic and systemic diseases of aniridia subjects during long-term follow-up.

## Zusammenfassung

**Hintergrund** Die kongenitale Aniridie ist eine schwere Fehlbildung fast aller Augensegmente. Darüber hinaus können auch endokrinologische, metabolische und zentralnervöse Erkrankungen vorliegen. Um bessere Behandlungsmöglichkeiten für diese seltene Krankheit zu entwickeln, ist die Einrichtung eines Aniridie-Zentrums notwendig. Unser Ziel ist es, die ophthalmologischen Befunde von Aniridiepatienten zusammenzufassen, die in der Klinik für Augenheilkunde am Universitätsklinikum des Saarlandes (UKS) in Homburg untersucht wurden.

**Methoden** Unsere retrospektive, monozentrische Studie umfasste Patienten, die sich zwischen Juni 2003 und Januar 2022 einer umfassenden augenärztlichen Untersuchung durch die Leiterin der Abteilung KiOLoN (Kinderophthalmologie, Orthoptik, Low Vision und Neuroophthalmologie) unterzogen. Die Daten des ersten Untersuchungszeitpunkts wurden einbezogen.

**Ergebnisse** Es wurden 556 Augen von 286 Probanden (20,1 ± 20,1 Jahre; 45,5% Männer) eingeschlossen. Bei 518 (93,7%) Augen lag ein Nystagmus vor, Strabismus bei 327 (58,8%) Augen. 436 (78,4%) Augen wiesen eine altersentsprechende Achsenlänge auf, 104 (18,7%) Augen hatten einen Mikrophthalmus und 13 (2,3%) Augen einen Buphthalmus. Bei 34 Augen (6,1%) lag eine Irisfehlbildung mit atypischem Kolobom vor, bei 61 Augen (10,9%) ein Irisrest von mehr als 6 Uhrzeiten, bei 96 Augen (17,2%) ein Irisrest von weniger als 6 Uhrzeiten und bei 320 Augen (57,5%) eine vollständige Aniridie. Die Patienten wurden nach den folgenden Stadien der aniridieassoziierten Keratopathie (AAK) eingeteilt: Stadium 0 (96 Augen [17,2%], keine Keratopathie), Stadium 1 (178 Augen [32,0%]), Stadium 2 (107 Augen 819,2%]), Stadium 3 (67 Augen [12,0%9), Stadium 4 (62 Augen [11,1%]), Stadium 5 (45 Augen [8,0%]). Ein sekundäres Glaukom lag bei 307 (55,5%), eine Makulahypoplasie bei 395 (71,4%) und eine angeborene Pathologie des Sehnervenkopfes bei 223 (40,3%) Augen vor. Die Art der Irisfehlbildung war signifikant positiv korreliert mit dem AAK-Stadium, den Linseneigenschaften, dem Vorhandensein eines Glaukoms, den angeborenen Makula- und Sehnervenkopf-Eigenschaften (p < 0,001 für alle), wobei eine komplette Aniridie die meisten Komplikationen aufwies.

**Schlussfolgerungen** Im Homburger Aniridiezentrum waren die häufigsten ophthalmologischen Zeichen bei kongenitaler Aniridie AAK, Irisfehlbildung, Katarakt und Makulahypoplasie. Der Typ der Irisfehlbildung kann auf die zukünftige Ausprägung von AAK, Katarakt und Glaukom hinweisen und ist mit der angeborenen Pathologie des Sehnervenkopfes und der Makula korreliert. Unser Register wird eine weitere detaillierte Analyse der ophthalmischen und systemischen Erkrankungen von Aniridie-Patienten im Langzeitverlauf ermöglichen.

## Introduction

Aniridia is considered a rare disease, with a global prevalence of 1 in 40 000 to 1 in 100 000 1 – 5 . Despite its name, aniridia is a panocular disorder that takes its name from the obvious hypoplasia of the iris, which is present in most cases. This feature can range from a conspicuous, almost complete loss of the iris to enlargement and irregularity of the pupil, representing a coloboma, to microscopic slit-like anomalies of the pupillary margin that can only be seen with slit lamp illumination. The effects on vision are also variable. In most cases, there is already congenital severe visual loss and, consequently, pathological visual development and nystagmus 1 , 2 , 3 , 4 , 6 .

In congenital aniridia, one can distinguish between the PAX6 gene-associated forms and other forms without alterations in the PAX6 gene, with the PAX6 forms being significantly more common 7 , 8 . The typical clinical PAX6-related congenital aniridia occurs in several forms: dominant inheritance, occurring sporadically (then inherited dominantly), as part of the WAGR (Wilms tumor, aniridia, genitourethral anomalies, retardation) or WAGRO (WAGR plus “obesity”) syndrome, and associated with other syndromes. Long-term complications with visual impairment, such as glaucoma or severe aniridia-associated keratopathy (AAK), are more frequent in PAX6-related aniridia 1 , 3 .

Since it has become more and more evident in recent years that so-called “isolated” PAX6 aniridia can also frequently have systemic concomitant diseases (hormonal, gastrointestinal, metabolic, cerebral), the term “aniridia syndrome” or “PAX6 syndrome” has been recommended 1 , 3 .

In order to develop better treatment options for the rare disease congenital aniridia, establishment of an aniridia center is necessary. The purpose of this work is to summarize ophthalmic properties of aniridia subjects examined at the Department of Ophthalmology, Saarland University Medical Center, in Homburg/Saar, Germany.

## Patients and Methods

### Ethical considerations

Our retrospective single-center study included patients from the Department of Ophthalmology, Saarland University Medical Center in Homburg/Saar, Germany. This study was approved by the Ethics Committee of Saarland/Germany (No 144/15) and followed regulations of the Declaration of Helsinki. Informed consent was obtained from all participants. In case of minors or guardianship, informed consent was obtained from the legal representative or legal guardian.

### Inclusion criteria, data collection, and examination methods

Inclusion criteria was the presence of partial or complete congenital aniridia, visible at slit lamp examination. All subjects underwent a structured ophthalmic examination through the Head of the KiOLoN (“Kinderophthalmologie”, Orthoptics, Low Vision and Neuroophthalmology) Unit of the Department of Ophthalmology of Saarland University, Prof. Dr. Barbara Käsmann-Kellner. Uncorrected and best-corrected visual acuity (UCVA and BCVA) measurement using Snellen charts, intraocular pressure (IOP) measurement using Goldmann applanation tonometry or iCare (Icare Finland Oy, Vantaa, Finland), and a detailed slit lamp and fundus examination were performed.

Iris malformation was classified as atypical coloboma, more than 6 clock hours of iris remnants, less than 6 clock hours of iris remnants, and complete aniridia (no iris remnant tissue is visible at slit lamp examination, without gonioscopy). Limbal stem cell insufficiency (LSCI) was classified as follows: (1) no limbal changes, (2) avascular pannus with less than 3 mm width, (3) vascularized pannus with less than 3 mm width, (4) vascularized pannus over 3 mm width. AAK was classified as Stage 0 (no limbal changes), Stage 1 (conjunctival tissue just crosses the limbal border but remains 1 mm or less from the limbus), Stage 2 (the pannus extends across the peripheral cornea and is typically present in 360 degrees of the cornea), Stage 3 (the pannus invades the central cornea, typically covering the entire cornea with vessels), Stage 4 (the cornea is completely vascularized), or Stage 5 (end-stage with an opaque, thick, vascularized cornea) 7 , 8 .

All patient data were entered pseudonymized in a Microsoft Access database. In collaboration with the Department of Ophthalmology, Saarland University Medical Center in Homburg/Saar (Chair: Prof. Dr. B. Seitz) and the Dr. Rolf M. Schwiete Center for Limbal Stem Cell and Aniridia Research, Homburg/Saar (Chair: Prof. Dr. N. Szentmáry), our aim was to build up a database in order to get better insight into the pathomechanisms and stage-appropriated treatment options of congenital aniridia. The present study summarizes patient data at the first examination time point for subjects examined between June 2003 and January 2022.

## Results

**Fig. 1:**
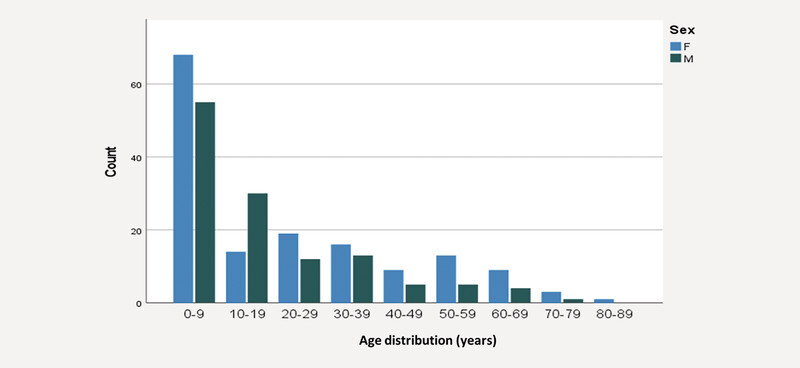
Age distribution of subjects at the Homburg Aniridia Center at the first examination time point.

**Fig. 2:**
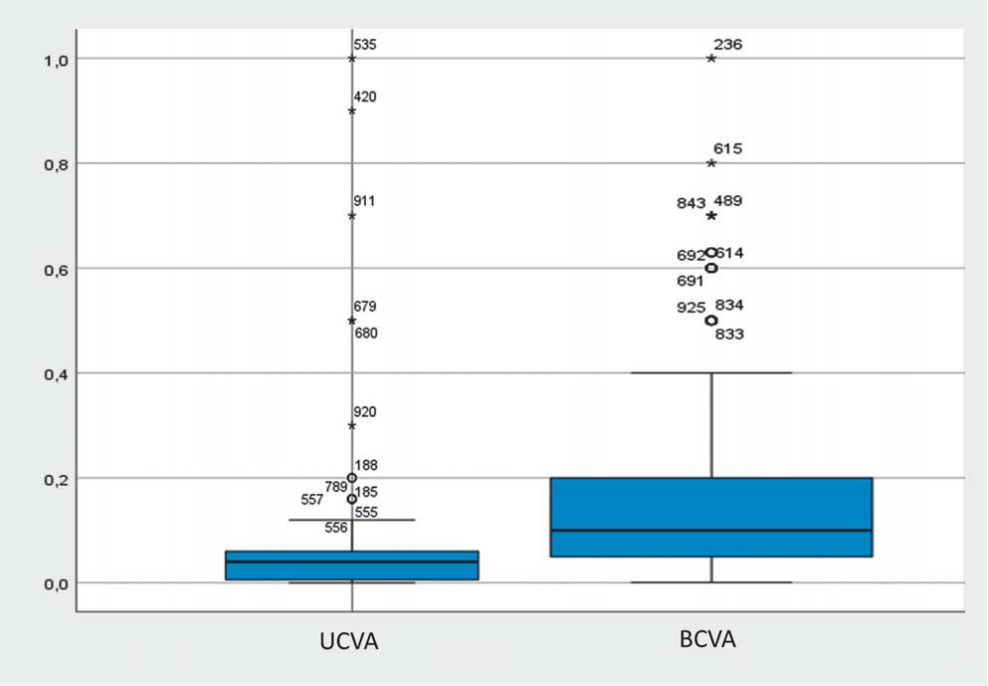
Uncorrected visual acuity (UCVA) and best-corrected visual acuity (BCVA; Snellen) of the analyzed subjects at the first examination time point.

Of 286 subjects, 556 eyes (age 20.1 ± 20.1 years; 45.5% males) were included. Age distribution of the subjects is displayed in Fig. 1 . UCVA was 0.074 ± 0.013 (0.001 – 1.0) and BCVA was 0.15 ± 0.08 (0.001 – 1.0; Fig. 2 ) at the first examination time point.

**Fig. 3:**
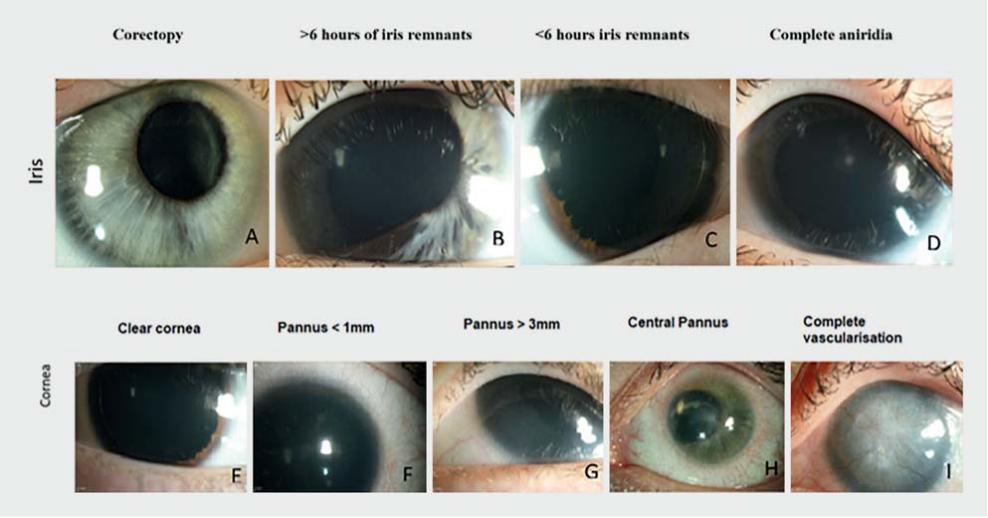
Iris malformation ( **a** – **d** ) and aniridia-associated keratopathy ( **e, f, g, h** ) in congenital aniridia subjects.

There was nystagmus in 518 (93.7%) eyes and strabismus in 327 (58.8%) eyes. There were 436 (78.4%) eyes with age-appropriate axial length, 104 (18.7%) eyes with microphthalmos, 13 (2.3%) eyes with buphthalmos, and in 3 (0.6) eyes, no axial length measurement was performed at the first examination time point. There was iris malformation with atypical coloboma in 34 eyes (6.1%), more than 6 clock hours of iris remnants in 61 eyes (10.9%), less than 6 clock hours of iris remnants in 96 eyes (17.2%), and complete aniridia in 320 (57.5%) eyes ( Fig. 3 a – d ). Nevertheless, in 45 (8.3%) eyes, we could not collect data on the exact iris malformation type, mainly due to corneal opacities.

LSCI was classified as follows: (1) no limbal changes in 97 eyes (17.4%), (2) avascular pannus with less than 3 mm width in 174 eyes (31.2%), (3) vascularized pannus with less than 3 mm width in 79 eyes (14.2%), and (4) vascularized pannus over 3 mm width in 190 eyes (34.1%). In 16 eyes (3.1%), no data on LSCI was available at the first time point of examination.

There was AAK Stage 0 (no keratopathy) in 96 eyes (17.2%), Stage 1 in 178 eyes (32.0%) eyes, Stage 2 in 107 eyes (19.2%) eyes, Stage 3 in 67 eyes (12.0%) eyes, Stage 4 in 62 eyes (11.1%), and Stage 5 in 45 eyes (8.0%). One eye (0.3%) could not be included in any of the groups along the available clinical data ( Fig. 3 e – i ).

**Fig. 4:**
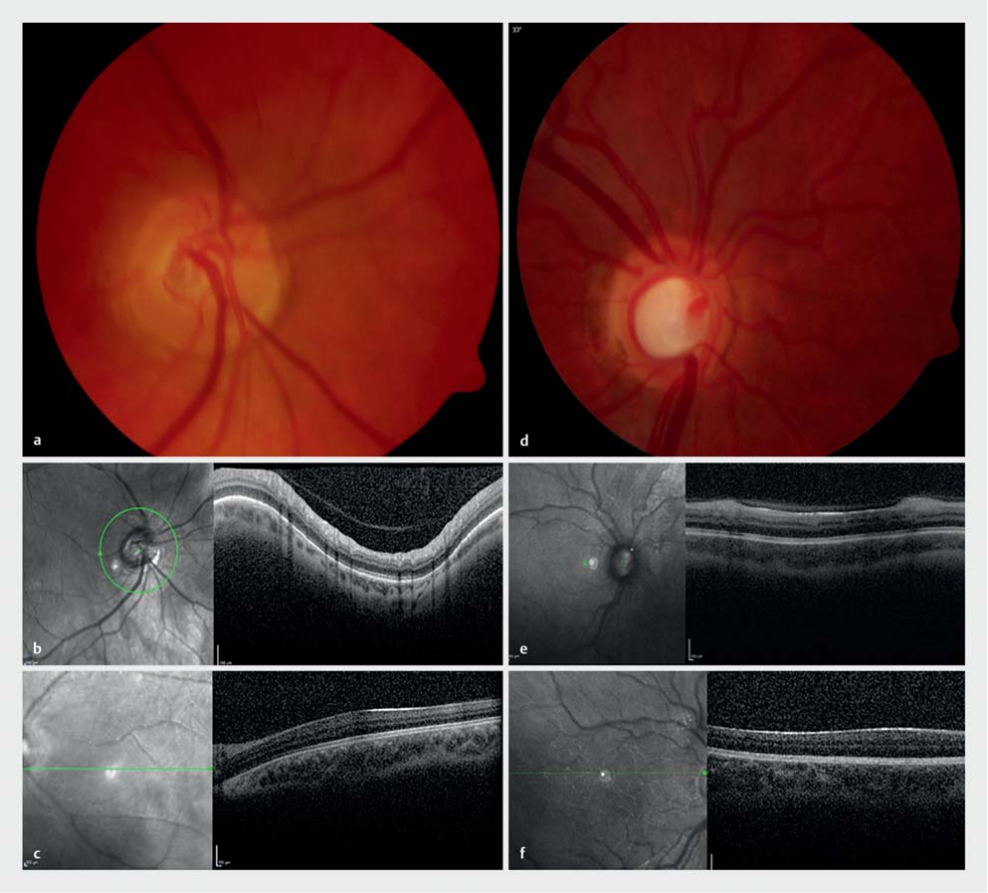
Congenital optic nerve head pathology ( **a** , **b** ) and macular hypoplasia ( **c** ), glaucomatous excavation ( **d** , **e** ), and macular hypoplasia ( **f** ) in congenital aniridia.

The lens was clear in 127 eyes (22.8%), there was cataract in 224 eyes (40.2%), subluxated lens in 9 eyes (1.6%), pseudophakia in 129 eyes (23.2%), aphakia in 32 eyes (5.7%), and in 35 eyes (6.5%), the lens status could not be assessed. There was secondary glaucoma in 307 eyes (55.5%), macular hypoplasia in 395 eyes (71.4%), and congenital optic nerve head pathology in 223 eyes (40.3%) eyes ( Fig. 4 ).

The iris malformation type was significantly positively correlated with AAK stage, lens properties, presence of glaucoma, congenital macular, and optic nerve head properties (p < 0.001 for all), with complete aniridia showing the most complications.

## Discussion

Collecting data of 556 eyes of 286 subjects from one of the largest worldwide aniridia databases, the Homburg Aniridia Center could be established in Homburg/Saar. About one-fourth of the included subjects were children. With a mean UCVA below 0.1 and a mean BCVA below 0.2, most of the analyzed subjects necessitated special education and visual aids during life 1 , 2 , 3 .

The misdevelopment of the iris is the characteristic phenotypic appearance in aniridia. It can range from a complete absence of the iris to a slight shift of the pupil (corectopy) or an atypical coloboma 7 , 8 . Iris malformation is one of the causes of photophobia. We could observe that about two-thirds of the analyzed subjects (75.3%) had less than 6 clock hours of iris remnants (17.4%), or complete aniridia (in 320 eyes [57.9%]), which enables a relatively obvious immediate diagnosis for ophthalmologists. Nevertheless, ophthalmologists also have to take into consideration that about 17% of the patients with congenital aniridia may present with an atypical coloboma (6.1%), or more than 6 clock hours of iris remnants (11%), but these signs may also indicate congenital aniridia. Additionally, in some cases, due to corneal opacities, the lack of iris might not be observable, which may result in a wrong diagnosis.

Most interestingly, statistical analysis confirmed the clinical suspicion that the iris malformation type was significantly positively correlated with AAK stage, lens properties, presence of glaucoma, congenital macular, and optic nerve head properties (p < 0.001 for all), with complete aniridia showing the most complications.

Up to 70% of PAX6 aniridia sufferers develop AAK with age, which may be due to a combination of several factors 1 – 3 , 6 , 7 , 8 , 9 , 10 . These include a pronounced dry eye problem, LSCI with impaired corneal epithelial cell differentiation, abnormal cell adhesion, and wound healing 1 , 2 , 3 , 5 – 10 . The consequences for the patients are permanent tear film instability as well as recurrent extremely painful corneal erosions, and a progression of visual loss due to vascularized corneal pannus and/or scars 5 , 7 – 9 . Among our subjects, with relatively young age, most of the subjects belonged to the Stage 1 AAK group (38.8% of the eyes), followed by Stage 2 with 19.3%, Stage 0 with 17.4%, Stage 3 in 12.1%, Stage 4 in 11.2%, and Stage 5 in 8.1% of the eyes.

AAK is characterized by centripetal spreading vascularization, conjunctivalization, and thickening of the cornea, which is, in part, due to LSCI 5 , 10 , 11 , 12 , 13 , 14 . There was LSCI with avascular pannus with less than 3 mm width in 174 (31.2%) eyes, vascularized pannus with less than 3 mm width in 79 (14.2%) eyes, and vascularized pannus over 3 mm width in 190 (34.1%) eyes.

The natural history of AAK shows several stages of progression. Signs of keratopathy often appear in early youth with thickening of the peripheral corneal epithelium but no functional manifestation. In the second decade, patients show chronic irritation and thin superficial vascularization in the peripheral cornea, which gradually progresses to the central cornea. Pain, photophobia, and recurrent corneal epithelial erosions are common. In later stages, the keratopathy progresses until the entire cornea is involved, with a severe increase in central corneal thickness due to pannus formation and vascularized scars 5 , 7 , 9 .

Cataract occurs in 50 – 85% of aniridia patients. In the Homburg Aniridia Center, there was a clear lens in 127 eyes (23.0%), cataract in 224 eyes (40.6%), subluxated lens in 9 eyes (1.6%), pseudophakia in 129 eyes (23.3%), aphakia in 32 eyes (5.8%), and in 21 eyes (3.8%), the lens status could not be assessed. In many cases, a cataract of the anterior and posterior lens pole (cataracta polaris anterior or posterior) is found congenitally, which often remains stable during life. In addition, progressive opacification of the other lens segments may occur, as well as subluxation or luxation of the crystalline lens due to lack/insufficiency of zonular fibers. Both may be an indication for lens removal and if possible, implantation of an intraocular lens to preserve visual acuity 1 – 3 , 6 , 9 .

Secondary glaucoma occurs with an incidence of 6 – 75% in aniridia subjects, often before adulthood. There was secondary glaucoma in 307 eyes (55.5%) of our subjects. Aniridia-associated glaucoma is caused by an abnormal localization of the Schlemm canal or by iris rudiments, which close the chamber angle or the trabecular meshwork and thus obstruct the outflow of aqueous humor. Diagnosis requires regular monitoring of IOP as well as the optic nerve. Often, a pressure measurement is difficult due to the thickened cornea and requires a corneal thickness measurement. Secondary glaucoma can lead to irreversible visual loss, even ending up in blindness due to glaucomatous optic atrophy and thus must be potentially considered the most serious irreversible complication 1 , 2 , 3 , 6 .

The Homburg Aniridia Center examined 395 (71.4%) eyes with macular hypoplasia and 223 (40.3%) eyes with congenital optic nerve head pathology. In aniridia patients, hypoplasia of the optic nerve and macula was more common. Macular hypoplasia does not necessarily occur together with optic hypoplasia, it can also occur as an isolated symptom in the context of congenital visual impairment. Furthermore, a mostly horizontal nystagmus as well as strabismus can be observed. We observed nystagmus in 518 (93.7%) eyes and strabismus in 327 (58.8%) eyes.

With regard to therapy, there are currently no generally accepted treatment modalities. In case of corneal involvement (AAK), different therapeutic options arise depending on the severity, which range from autologous serum eye drops, amniotic membrane transplantation, and phototherapeutic keratectomy to lamellar and penetrating keratoplasty. For penetrating keratoplasties, the following procedure has been proven helpful in our Department of Ophthalmology in these high-risk patients: systemic immunosuppression, small-sized penetrating keratoplasty with single knot sutures, simultaneous transplantation of an amniotic membrane as a patch, temporary lateral tarsorrhaphy, and autologous serum eye drops 9 .

As treatment for limbal stem cell deficiency in congenital aniridia, the use of limbal allografts (4 eyes), keratolimbal allografts (31 eyes), cultivated limbal epithelial cells (10 eyes), and cultivated oral mucosal epithelial cells (17 eyes) have been reported 15 . These procedures may be combined with systemic immunosuppression, simultaneous transplantation of an amniotic membrane as a patch, temporary lateral tarsorrhaphy, and postoperative use of autologous serum eye drops. Following surgery, visual acuity improves during the first 6 months, which thereafter, gradually declines. Patients were followed for 12 – 18 months after epithelial (stem) cell transplantation, however, longer term outcomes and further procedures have not been reported, yet 1 – 4 , 9 , 14 , 15 .

Because of the feared aniridia-fibrosis syndrome and the increased risk of glaucoma due to obstruction of the chamber angle and thus of the aqueous humor outflow, the surgical insertion of an artificial iris should be avoided. Anti-glaucomatous therapy should be started before visual field loss occurs. If an IOP increase is resistant to therapy, trabeculotomy is the method of first choice. Lens opacification can be surgically treated by insertion of an artificial lens, if necessary, with a capsular tension ring (small incision, “in-the-bag”), if visual acuity is clearly impaired 1 , 2 , 3 , 4 , 9 .

Prognosis limiting is the observation that surgical therapy options in aniridia patients have a multiple higher complication rate than in patients not affected by this disease. One well-known complication after repeated ocular surgery is progressive anterior segment fibrosis syndrome. In this case, a non-acute inflammatory fibrotic membrane develops in the anterior chamber starting from the iris remnants, which grows into the posterior chamber over the ciliary body, detaching the ciliary body and thus causing bulbar hypotony, with consecutive retinal detachment 1 , 2 , 3 , 4 , 9 .

Due to the rarity of aniridia, all ophthalmic clinics in Germany, university based or others, care for relatively few patients. Thus, no sound treatment guidelines exist for PAX6 aniridia and its complications. We established a clinical aniridia registry (observational study) at our hospital in order to systematically record the course of this rare disease, to optimize its diagnostics and therapy, and thus to further improve the treatment of the disease in the future.

Currently, 460 patients with congenital aniridia are regularly followed at our clinic (2/3 are children and adolescents under 16 years of age, and 1/3 are adults over 16 years), more than 98% of whom live outside Saarland. Nevertheless, it is estimated that there are currently about 940 patients in Germany with the diagnosis “congenital aniridia” (about 85% may be PAX6-associated aniridia). Most of these patients or their parents or legally designated caregivers have registered themselves in the national self-help organization “AWS Aniridie-WAGR e. V.” ( www.aniridie-wagr.de ).

Prof. Käsmann-Kellner has been a volunteer medical advisor to the association since its inception, and the association supported the establishment of our Aniridia Center. A large proportion of the patients we care for are children, and accordingly – as with most of the rare congenital diseases – measurable effects of our planned interventions may not become apparent for many years. The aim is therefore to store the data collected in the registry for the long term, initially for 10 years, allowing a longitudinal long-term follow-up.

Conclusion Box **Already known:**

- In congenital aniridia, there is an increased risk of developing blindness during life.
- As congenital aniridia is a rare disease, it is difficult to establish clinical prognostic parameters and treatment standards.
- In order to develop better treatment options in congenital aniridia, establishment of an aniridia center is necessary.

**Newly described:**

- The most prevalent ophthalmic signs in congenital aniridia are AAK, iris malformation, cataract, and macular hypoplasia.
- The iris malformation type may indicate future expression of AAK, cataract, and glaucoma development and it is correlated with congenital optic nerve head and macular pathology.
- Our Aniridia Center will support further detailed longitudinal analysis of ophthalmic and systemic diseases of these difficult patients with congenital aniridia during long-term follow-up.

## References

[1] Käsmann-Kellner B Fries F N Latta L Aniridie bei PAX6-Syndrom: Eine potenziell zur Erblindung führende Erkrankung. Etablierung eines Deutschen Aniridie-Registers. Concept Ophthalmologie 2019; 12–18 https://www.researchgate.net/publication/353515480_Aniridie_bei_PAX6-Syndrom_Eine_potenziell_zur_Erblindung_fuhrende_Erkrankung_Etablierung_eines_Deutschen_Aniridie-Registers

[2] Käsmann-Kellner B Seitz B [Aniridia syndrome: clinical findings, problematic courses and suggestions for optimization of care (“aniridia guide”)] Ophthalmologe 2014 111 1145 1156 10.1007/s00347-014-3060-x 25475188

[3] Käsmann-Kellner B Seitz B [Congenital aniridia or PAX6 syndrome] Ophthalmologe 2014 111 1144 10.1007/s00347-014-3058-4 25475186

[4] Seitz B Käsmann-Kellner B Viestenz A [Stage-related therapy of congenital aniridia] Ophthalmologe 2014 111 1164 1171 10.1007/s00347-014-3061-9 25475189

[5] Latta L Figueiredo F C Ashery-Padan R Pathophysiology of aniridia-associated keratopathy: Developmental aspects and unanswered questions Ocul Surf 2021 22 245 266 10.1016/j.jtos.2021.09.001 34520870

[6] Käsmann-Kellner B Latta L Fries F N Diagnostic impact of anterior segment angiography of limbal stem cell insufficiency in PAX6-related aniridia Clin Anat 2018 31 392 397 10.1002/ca.22987 28906020

[7] Lagali N Wowra B Fries F N PAX6 Mutational Status Determines Aniridia-Associated Keratopathy Phenotype Ophthalmology 2020 127 273 275 10.1016/j.ophtha.2019.09.034 31708273

[8] Lagali N Wowra B Fries F N Early phenotypic features of aniridia-associated keratopathy and association with PAX6 coding mutations Ocul Surf 2020 18 130 140 10.1016/j.jtos.2019.11.002 31734509

[9] Farah C J Fries F N Latta L An attempt to optimize the outcome of penetrating keratoplasty in congenital aniridia-associated keratopathy (AAK) Int Ophthalmol 2021 41 4091 4098 10.1007/s10792-021-01982-z 34324101 PMC8572819

[10] Katiyar P Stachon T Fries F N Decreased FABP5 and DSG1 protein expression following PAX6 knockdown of differentiated human limbal epithelial cells Exp Eye Res 2022 215 108904 10.1016/j.exer.2021.108904 34954205

[11] Latta L Knebel I Bleil C Similarities in DSG1 and KRT3 Downregulation through Retinoic Acid Treatment and PAX6 Knockdown Related Expression Profiles: Does PAX6 Affect RA Signaling in Limbal Epithelial Cells? Biomolecules 2021 11 1651 10.3390/biom11111651 34827649 PMC8615883

[12] Latta L Ludwig N Krammes L Abnormal neovascular and proliferative conjunctival phenotype in limbal stem cell deficiency is associated with altered microRNA and gene expression modulated by PAX6 mutational status in congenital aniridia Ocul Surf 2021 19 115 127 10.1016/j.jtos.2020.04.014 32422284

[13] Latta L Nordström K Stachon T Expression of retinoic acid signaling components ADH7 and ALDH1A1 is reduced in aniridia limbal epithelial cells and a siRNA primary cell based aniridia model Exp Eye Res 2019 179 8 17 10.1016/j.exer.2018.10.002 30292490

[14] Schlötzer-Schrehardt U Latta L Gießl A Dysfunction of the limbal epithelial stem cell niche in aniridia-associated keratopathy Ocul Surf 2021 21 160 173 10.1016/j.jtos.2021.06.002 34102310

[15] Landsend E CS Lagali N Utheim T P Congenital aniridia – A comprehensive review of clinical features and therapeutic approaches Surv Ophthalmol 2021 66 1031 1050 10.1016/j.survophthal.2021.02.011 33675823

